# Role of CD14 in a Mouse Model of Acute Lung Inflammation Induced by Different Lipopolysaccharide Chemotypes

**DOI:** 10.1371/journal.pone.0010183

**Published:** 2010-04-16

**Authors:** Adam A. Anas, Joppe W. R. Hovius, Cornelis van 't Veer, Tom van der Poll, Alex F. de Vos

**Affiliations:** Center for Infection and Immunity Amsterdam, and Center for Experimental and Molecular Medicine, Academic Medical Center, University of Amsterdam, Amsterdam, The Netherlands; Hannover School of Medicine, Germany

## Abstract

**Background:**

Recognition of lipopolysaccharide (LPS) is required for effective defense against invading gram-negative bacteria. Recently, *in vitro* studies revealed that CD14 is required for activation of the myeloid differentiation factor (MyD)88-dependent Toll-like receptor (TLR)4 signaling pathway by smooth (S)-LPS, but not by rough (R)-LPS. The present study investigated the role of CD14 in induction of lung inflammation in mice by these different LPS chemotypes.

**Methodology/Results:**

Neutrophil accumulation and tumor necrosis factor (TNF) release in bronchoalveolar lavage fluid were determined 6 hours after intranasal treatment of wild type (WT) and CD14 knock-out (KO) mice with different doses S-LPS or R-LPS. The contribution of CD14 to lung inflammation induced by S-LPS or R-LPS depended on the LPS dose. At low doses, S-LPS and R-LPS induced neutrophil influx in a CD14-dependent manner. Low dose S-LPS-induced cytokine release also depended on CD14. Strikingly, neutrophil influx and TNF release induced by high dose S-LPS or R-LPS was diminished in the presence of CD14. Intranasal administration of sCD14 to CD14 KO mice treated with S-LPS partially reversed the inflammatory response to the response observed in WT mice.

**Conclusions:**

In conclusion, CD14 modulates effects of both S-LPS and R-LPS within the lung in a similar way. Except for R-LPS-induced TNF release, S-LPS and R-LPS at low dose induced acute lung inflammation in a CD14-dependent manner, while the inflammatory response triggered by high dose S-LPS or R-LPS was diminished by CD14.

## Introduction

Recognition of endotoxin or LPS, a major constituent of the outer membrane of gram-negative bacteria, has been extensively studied to clarify the mechanisms by which this component activates the immune system [1]. Detection of LPS and initiation of a rapid inflammatory response are required for effective defense against invading gram-negative bacteria [2].

LPS is bound by MD-2 within the TLR4/MD-2 complex [3] and subsequent conformational changes in TLR4 lead to reorganization of its cytoplasmic domain, enabling the recruitment of the adaptors MyD88 and Toll/interleukin 1 receptor domain-containing adaptor inducing interferon beta (TRIF) [4]. These adaptors initiate signal transduction to the nucleus leading to production of cytokines and chemokines that regulate inflammatory cells [4]. Binding of LPS to the TLR4/MD-2 complex is facilitated by LPS binding protein (LBP) and CD14 [1]. LBP, which is present in the bloodstream and the lung [5], [6], binds to LPS aggregates and transfers LPS monomers to CD14 [7]. CD14, a 55-kDa glycoprotein predominantly expressed on the surface of myeloid cells via a glycosylphosphatidyl anchor, associates with the TLR4/MD-2 complex and transfer LPS monomers to TLR4/MD-2 [8]. CD14 also exists in a soluble form (sCD14), which is able to mediate LPS-activation of cells devoid of membrane CD14 expression, such as epithelial and endothelial cells [9]. However, high concentrations of sCD14 may interfere with LPS-induced activation of CD14-expressing cells like macrophages [10], [11].

LPS synthesized by most gram-negative bacteria consists of three modules, the lipid A moiety, a core polysaccharide and an O-polysaccharide of variable length (consisting of 1 to 50 monosaccharide units)[12], [13] and is designated smooth LPS (S-LPS). Gram-negative bacteria that fail to add the core polysaccharide or the O-polysaccharide chain to lipid A produce ‘rough’ LPS (R-LPS). Lipid A, the bioactive part of both S-LPS and R-LPS, is responsible for most of the pathogenic effects in gram-negative bacterial infections [1].

Recently, it was reported that in the absence of CD14, the TLR4/MD-2 complex can distinguish between these LPS chemotypes [14]. Macrophages lacking CD14 secreted equal amounts of TNF as macrophages expressing CD14 upon stimulation with R-LPS, but failed to secrete TNF in response to S-LPS which was reversed by addition of sCD14 [14]. These data indicate that the TLR4/MD-2 complex requires CD14 for the activation of MyD88-dependent signaling by S-LPS, but not by R-LPS. Previously, we and others showed that CD14 is an essential receptor in LPS-induced lung inflammation and pneumonia caused by gram-negative bacteria [15]–[18]. The aim of the present study was to investigate the role of CD14 in the induction of acute lung inflammation by these different LPS chemotypes.

## Results

### S-LPS- and R-LPS-induced lung inflammation is dependent on TLR4 and MyD88

**Figure 1:**
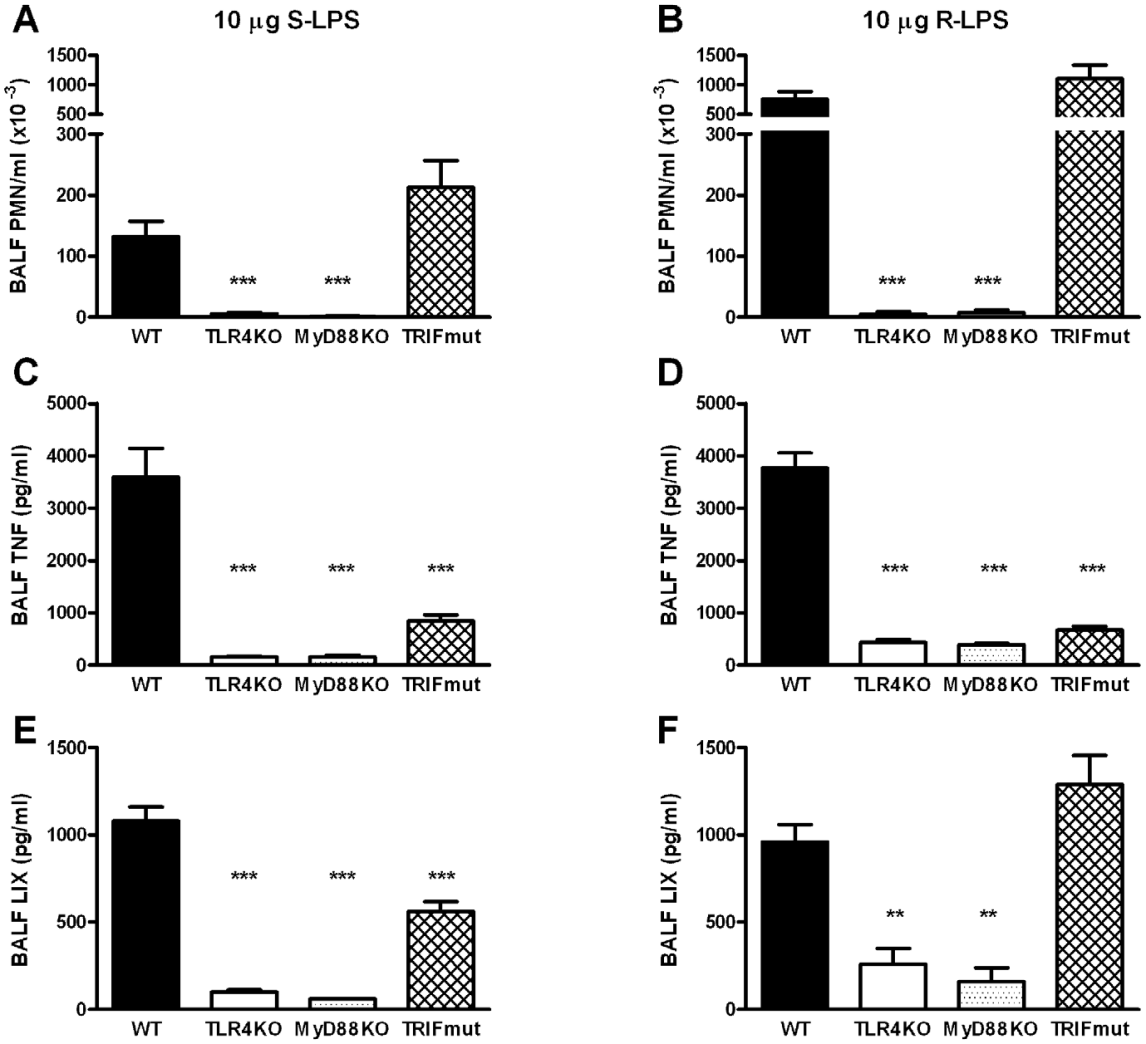
S-LPS- and R-LPS-induced acute lung inflammation is dependent on TLR4 and MyD88 and partially TRIF dependent. Mice (n = 8–9 per group) were inoculated intranasally with 10 µg of S-LPS (left panel) or R-LPS (right panel) and analysed 6 hours later for lung PMN influx (A, B), and TNF (C, D) and LIX (E, F) release in BALF. Data are mean ± SEM. **, P<0.01, *** P<0001 versus WT mice.

Previous studies have established that the pulmonary response to LPS totally relies on the presence of TLR4 [15], [18]. Considering that CD14 is a co-receptor within the TLR4 receptor complex, we first investigated whether S-LPS or R-LPS administered intranasally to mice also signals through TLR4. Additionally, MyD88KO and TRIFmut mice were treated with these LPS chemotypes in order to establish the TLR4 signaling pathways involved in this inflammation model. Thus, WT, TLR4KO, MyD88KO and TRIFmut mice were treated with 10 µg of S-LPS or R-LPS and the influx of polymorphonuclear cells (PMNs) into BALF, as well as the BALF concentrations of TNF (a cytokine primarily produced by macrophages)[19], [20] and LIX (a chemokine exclusively produced by respiratory epithelial cells)[20] was measured as read outs for the pulmonary response to local LPS instillation. BALF was obtained 6 hours after LPS administration, since this time point is representative for both PMN influx and local cytokine/chemokine release [6], [15], [18], [20]. Compared to WT mice, S-LPS- or R-LPS-induced PMN influx was equally and strongly reduced in TLR4KO and MyD88KO mice (P<0.001, Fig. 1A,B). Similarly, BALF TNF and LIX concentrations were markedly and equally reduced in TLR4KO and MyD88KO upon intrapulmonary delivery of S-LPS or R-LPS (P<0.01, Fig. 1 C–F). In TRIFmut mice, S-LPS- or R-LPS-induced BALF TNF levels were also strongly reduced (P<0.001), but PMN influx and BALF LIX levels were not or modestly lowered (Fig. 1A–F). These results indicate that the pulmonary response triggered by either S-LPS or R-LPS requires TLR4 and predominantly MyD88-dependent signaling.

### CD14 deficiency results in enhanced TNF release upon pulmonary instillation of S-LPS and R-LPS

**Figure 2:**
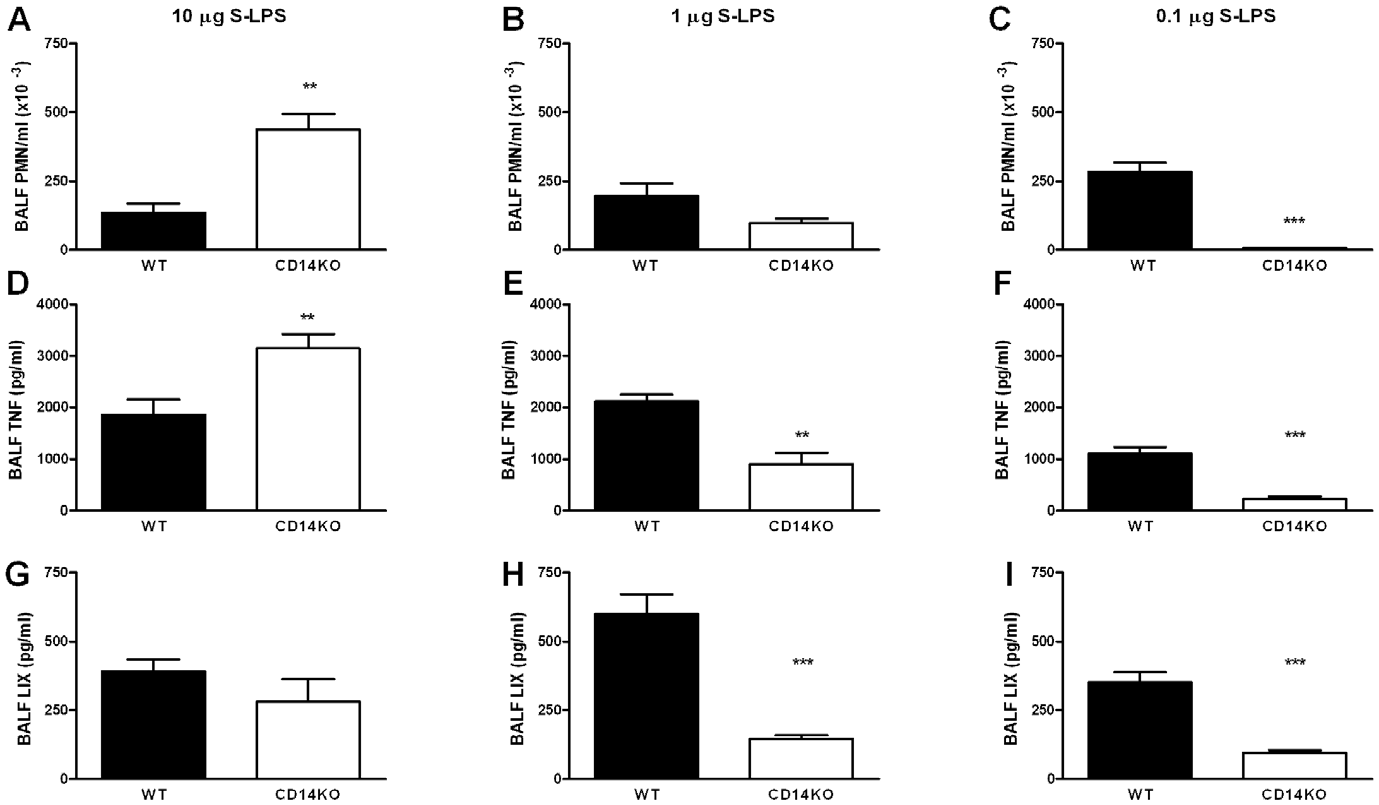
Pulmonary CD14 diminishes lung inflammation by high dose S-LPS, but enhances lung inflammation by low dose S-LPS. Mice (n = 7–9) were treated intranasally with 10 µg S-LPS (left panel), 1 µg S-LPS (middle panel) or 0.1 µg S-LPS (right panel). Six hours later BALF was isolated and analysed for PMN counts (A–C), TNF levels (D–F) and LIX levels (G–I). Data are mean ± SEM. **, P<0.01; ***, P<0001 versus WT mice.

**Figure 3:**
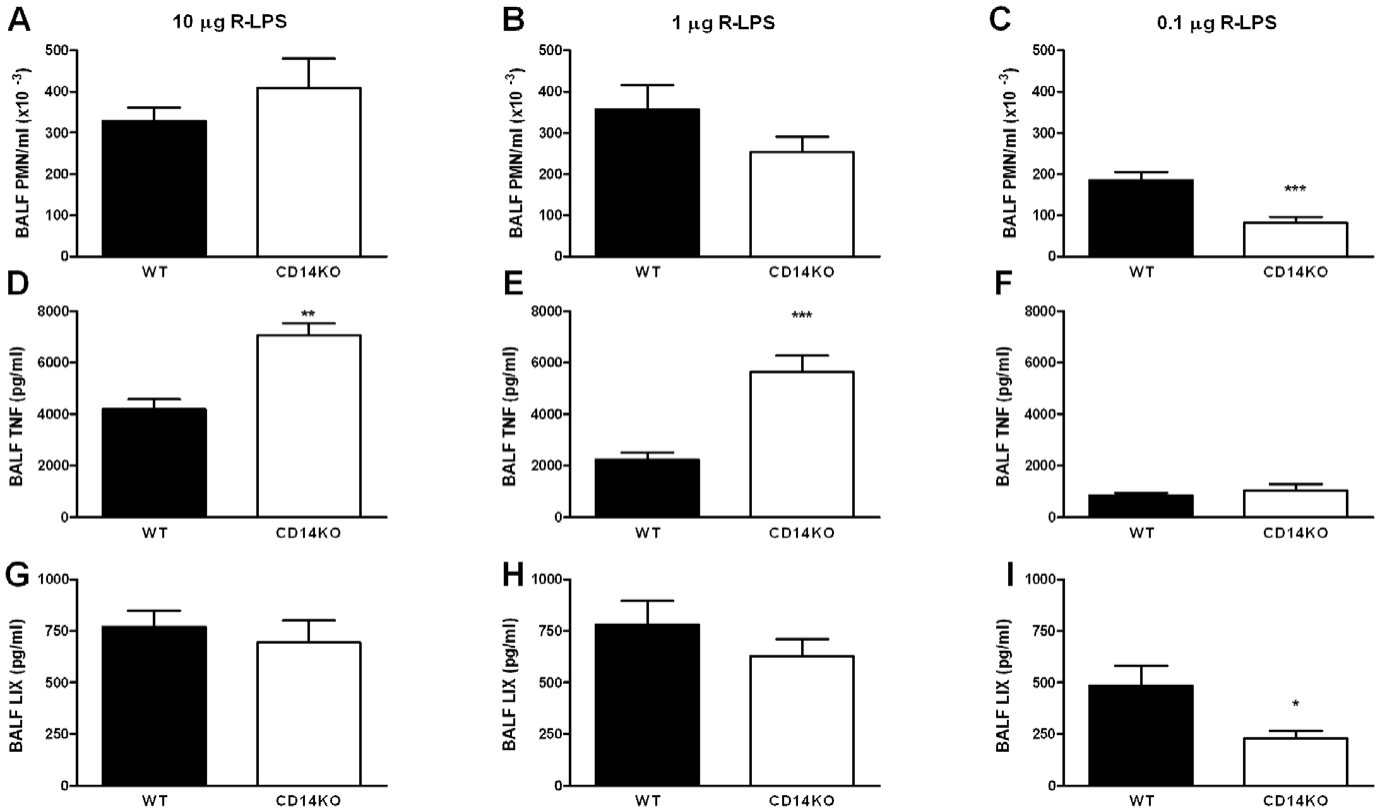
Pulmonary CD14 partially diminishes lung inflammation by high dose R-LPS, but enhances lung inflammation by low dose R-LPS. Mice (n = 6–9) were treated intranasally with 10 µg R-LPS (left panel), 1 µg R-LPS (middle panel) or 0.1 µg R-LPS (right panel). Six hours after LPS administration, BALF was isolated and analysed for PMN counts (A–C), TNF levels (D–F) and LIX levels (G–I). Data are mean ± SEM. *, P<0.05; **, P<0.01; ***, P<0001 versus WT mice.

To determine the role of CD14 in lung inflammation induced by S-LPS or R-LPS, WT and CD14KO mice were treated intranasally with 10 µg of either form of LPS and analyzed 6 hours later. Surprisingly, S-LPS induced a significantly higher PMN influx in CD14KO mice as compared to WT mice (P<0.01, Fig. 2A). In addition, 10 µg of S-LPS also induced higher concentrations of TNF (P<0.01) in BALF of CD14KO mice (Fig. 2D). R-LPS tended to elicit increased influx of PMNs in BALF of CD14KO mice (not significant, Fig. 3A), but did induce increased release of TNF (P<0.01, Fig. 3D). At this LPS dose, BALF LIX levels were not influenced by CD14 deficiency. Thus, inflammatory responses induced by either S-LPS or R-LPS within the bronchoalveolar space did not depend on CD14; in contrast, some responses were even enhanced in CD14KO mice, suggesting an inhibitory role of this receptor.

### CD14 deficiency attenuates lung inflammation induced by low doses of S-LPS or R-LPS

To determine whether the partially enhanced lung inflammation was dependent on the dose of LPS, WT and CD14KO mice were treated intranasally with lower amounts of S-LPS or R-LPS and analysed 6 hours later. CD14KO mice treated with 0.1 µg of LPS showed a reduced influx of PMNs in response to S-LPS or R-LPS (both P<0.001 versus WT mice, Fig. 2C and 3C). In response to 1 µg of either S-LPS or R-LPS, CD14KO mice tended to have an impaired PMN influx (not significant versus WT mice; Fig. 2B and 3B). This was accompanied by significantly reduced BALF TNF levels in S-LPS-treated CD14KO mice (P<0.01, Fig. 2E, F), but increased TNF levels in R-LPS-treated CD14KO mice (P<0.001, Fig. 3E). The local release of LIX was facilitated by the presence of CD14 at lower S-LPS and R-LPS doses, i.e. CD14KO mice treated with 0.1 µg of LPS displayed lower LIX BALF levels than WT mice (P<0.05, Fig 2I and 3I). Together, these findings reveal that CD14 in the lung either does not influence or diminishes inflammatory responses induced by high concentrations of S-LPS or R-LPS, but augments inflammation triggered by low concentrations of S-LPS or R-LPS. Moreover, CD14 does not facilitate local release of TNF induced by intrapulmonary R-LPS at any dose tested.

### Effects of sCD14 on S-LPS induced lung inflammation

**Figure 4:**
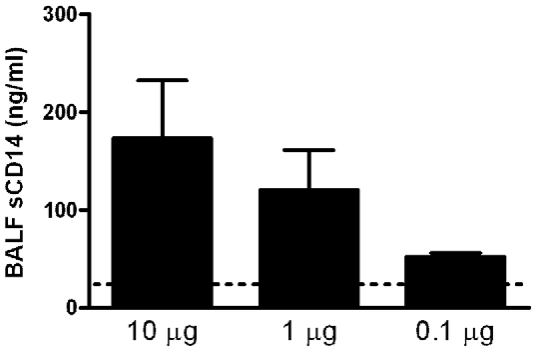
S-LPS induces sCD14 release in the lung in a dose dependent manner. sCD14 was measured in BALF obtained from WT mice 6 hours after intranasal administration of different doses (10–0.1 µg) of S-LPS. Eight to nine mice were used per group. Data are mean ± SEM. Dotted line represents the mean value of sCD14 in BALF of naive mice.

**Figure 5:**
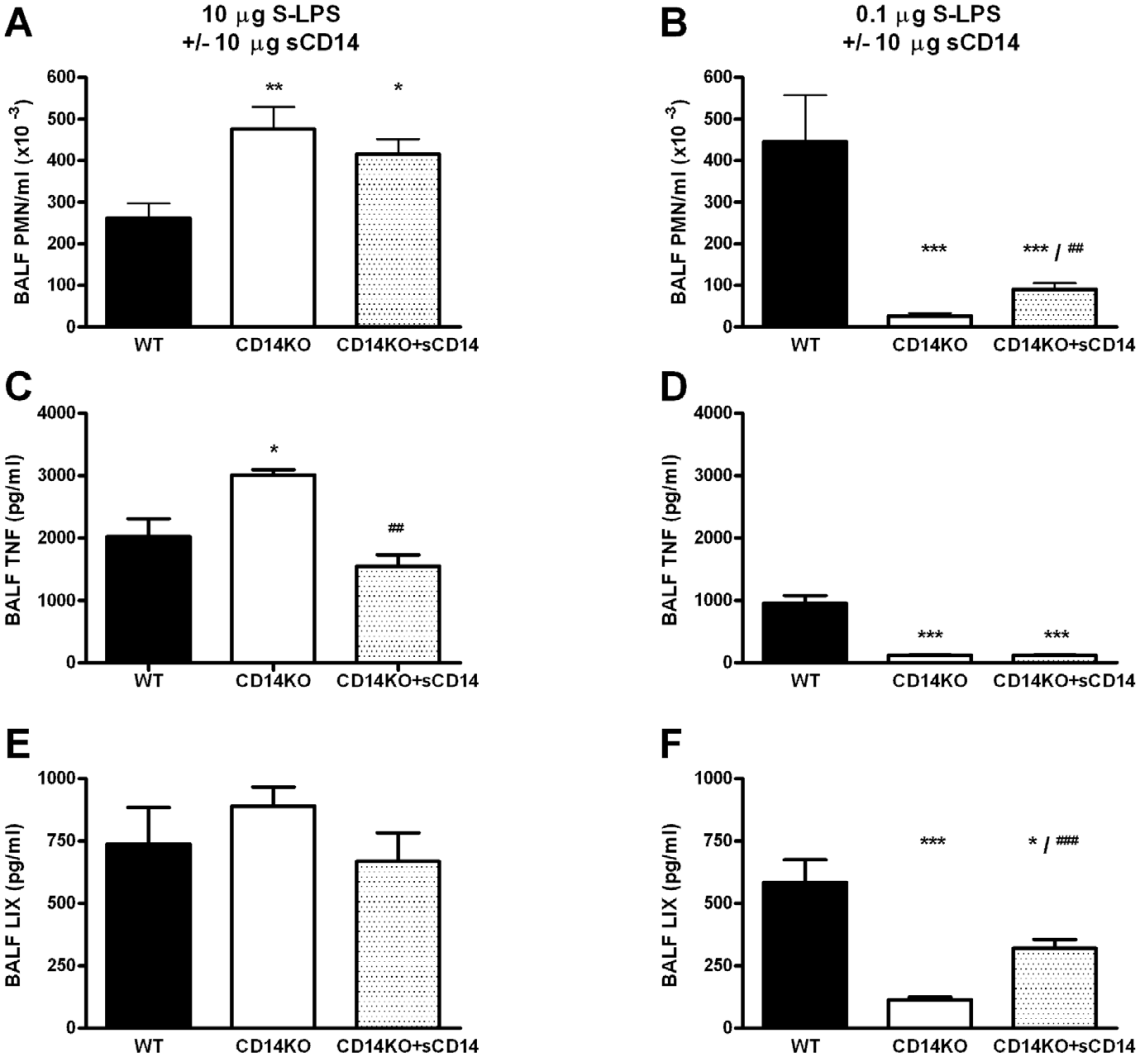
sCD14 exerts bimodal effects in acute lung inflammation depending on the dose of S-LPS. WT and CD14KO mice were treated intranasally with 10 µg S-LPS (left panel) or 0.1 µg S-LPS (right panel) and 10 µg sCD14 was administered simultaneously with S-LPS to groups of CD14KO mice. Six hours after LPS (and sCD14) administration, BALF was isolated and analyzed for PMN counts (A, B), TNF levels (C, D) and LIX levels (ER, F). Eight to nine mice were used per group. Data are are mean ± SEM. *, P<0.05; **, P<0.01; ***, P<0001 versus WT mice; ##, P<0.01; ###, P<0.001 versus CD14KO mice.

The data presented above provided clear evidence for a bimodal role of CD14 in the pulmonary responses induced by S-LPS. Since sCD14 can modulate LPS-induced responses [7], we were interested in establishing whether sCD14 can compensate for CD14 gene deficiency with regard to inhibition and enhancement of S-LPS effects at different doses. First, we measured sCD14 concentrations in BALF of WT mice 6 hours after instillation of different doses of S-LPS (10, 1 and 0.1 µg). As shown in figure 4, S-LPS elicited a dose-dependent rise in BALF sCD14 levels. To exclude the possibility that the increase in alveolar sCD14 levels resulted from leakage of serum proteins, total protein concentrations in BALF of LPS-treated WT mice were assessed. No differences in total BALF protein levels were observed in these mice 6 hours after treatment with 10, 1 or 0.1 µg S-LPS (data not shown). Next, we administered CD14KO mice with sCD14 (10 µg) intranasally together with S-LPS at either 10 µg (*i.e.* a dose at which CD14 inhibits S-LPS induced lung inflammation, Fig. 2) or 0.1 µg (*i.e.* a dose at which CD14 enhances S-LPS induced lung inflammation, Fig. 2). In these experiments the phenotype of CD14KO mice after intranasal administration of S-LPS at a high or low dose was reproduced (Fig. 5). Importantly, sCD14 treatment partially reversed the phenotype of CD14KO mice at both S-LPS doses. Specifically, whereas sCD14 did not impact on the enhanced PMN influx in CD14KO mice upon instillation of S-LPS at 10 µg (Fig. 5A), sCD14 reduced the exaggerated TNF release in CD14 KO mice to levels found in WT mice (P<0.01 for the difference with CD14 KO, Fig. 5C). At this LPS dose, neither sCD14 administration nor CD14 deficiency influenced LIX release. In addition, whereas sCD14 modestly but significantly increased the reduced PMN influx in CD14 KO mice upon instillation of S-LPS at 0.1 µg (P<0.01 for the difference with CD14 KO mice, Fig. 5B), this treatment did not influence the reduced TNF release into BALF in CD14 KO mice at this LPS dose (Fig. 5D). Remarkably, however, sCD14 administration strongly increased the release of LIX in CD14KO mice exposed to 0.1 µg S-LPS (P<0.001 versus CD14KO mice, Fig. 5F). Taken together, these results suggest that sCD14 may inhibit or facilitate S-LPS effects in the bronchoalveolar space depending on the LPS dose used.

## Discussion

CD14 was discovered as LPS receptor nearly two decades ago [1], but many aspects of its role in LPS-induced responses still remain to be clarified. Evidence derived from *in vitro* experiments has indicated that whereas the TLR4/MD-2 complex requires CD14 for the activation of MyD88-dependent signaling by S-LPS [14], the main form of LPS produced by most gram-negative bacteria, it does not require CD14 for activation by R-LPS. Elaborating on this notion, previous findings of us and others that CD14 is essential in LPS-induced lung inflammation [15]–[18] and pneumonia caused by *Acinetobacter baumannii* [15] are explained by the predominance of the smooth form of LPS in the inocula. Of note, however, *in vivo* experiments have revealed that the lethal effects of both S-LPS and R-LPS depend on CD14 [14]. Together, these findings prompted us to re-evaluate the role of pulmonary CD14 *in vivo* in acute lung inflammation induced by different LPS chemotypes. Using CD14KO mice treated intranasally with various doses of S-LPS or R-LPS, we demonstrate here that CD14 plays a bimodal role in the induction of PMN influx and local TNF release in response to intrapulmonary delivery of S-LPS, inhibiting S-LPS effects at high doses while facilitating the effects at low doses. Moreover, we show that sCD14 can partially reproduce these differential roles of CD14. In addition, our results reveal that CD14 modulates the effects of R-LPS and S-LPS within the lung in vivo in a similar way, with the important exception that this receptor did not facilitate TNF release at any R-LPS dose.

In the present study, we found at low doses that R-LPS (but not S-LPS) induced TNF secretion in the lung in a CD14-independent manner, whereas PMN recruitment into the lung was induced by these LPS chemotypes in a CD14-dependent manner. The requirement of CD14 in S-LPS-induced inflammatory responses is in line with previous *in vitro* and *in vivo* studies with cytokine release as read-out [12], [14]. Published data on the contribution of CD14 to R-LPS- induced cytokine release are inconsistent: CD14 has been reported to be irrelevant for R-LPS-induced TNF production [14], whereas other investigations found that CD14 augmented R-LPS-induced cytokine secretion by macrophages [12], [21] as well as plasma TNF levels triggered by intravenous R-LPS [12]. Our present results suggest that CD14 facilitates some but not all R-LPS-induced responses in the bronchoalveolar space. The contrasting influence of CD14 on TNF and PMN influx in the lung may result from differential CD14 dependency of lung cells responding to low dose R-LPS. Alveolar macrophages, which express both CD14 and TLR4 [22] and are major producers of TNF, may not require CD14 to respond to low dose R-LPS as previously found with peritoneal macrophages [14]. Lung epithelial cells, which constitutively express TLR4 but lack CD14 [23] and which are essential for the influx of PMN upon intrapulmonary instillation of LPS [24], may require (s)CD14 to respond to low doses of R-LPS [9], [12]. In accordance, CD14KO mice displayed lower BALF concentrations of LIX, a chemokine exclusively produced by respiratory epithelial cells [20], upon intranasal instillation of R-LPS at low doses.

At high doses, neither S-LPS nor R-LPS required CD14 to induce PMN influx or TNF and LIX secretion in BALF, which is in line with the results of others obtained with LPS stimulated macrophages [21] or a mouse model of LPS-induced lung inflammation [18]. Strikingly, in response to high dose LPS, PMN recruitment and TNF release in the lung were exaggerated in CD14KO mice relative to WT mice. Although our study does not elucidate the mechanism underlying this intriguing finding, we did demonstrate that high dose S-LPS (Fig. 4) and R-LPS (data not shown) induce the release of sCD14 in WT mice, which may down-regulate further LPS-induced inflammatory processes. Studies by Haziot *et al.* [10] and Stelter *et al.* [11] revealed that high concentrations of sCD14 can inhibit LPS-induced secretion of TNF by macrophages, which may result from transfer of LPS to lipoproteins and subsequent removal [7]. This notion is partially supported by our experiments in which sCD14 was administered to CD14KO mice, i.e. intranasal instillation of exogenous sCD14 together with high dose S-LPS to CD14KO mice resulted in a significant reduction of TNF release in the lung, but this treatment did not affect PMN infiltration into BALF. These findings suggest that sCD14 released in response to high dose LPS regulates LPS-responsiveness of cells secreting TNF, but not the cells responsible for the attraction of PMNs. This possibility is supported by the lack of an effect of (s)CD14 on LIX release after high dose LPS administration, considering that respiratory epithelial cells are important for both PMN influx and LIX secretion [20], [24]. Alternatively, high dose LPS in a CD14-dependent manner may trigger the release of LBP, which like sCD14 down-regulates LPS-induced inflammatory processes [7]. Previously, we found that lung inflammation induced by high dose LPS was enhanced in LBPKO mice [6], closely resembling the present findings in CD14KO mice. LBP levels in the lungs of WT and CD14KO mice treated with high dose LPS, however, did not differ (data not shown). Therefore, further investigations are required to determine the mechanism underlying the reduced inflammation in WT mice treated with high dose LPS as compared to CD14KO mice.

TLR4 induces two independent signaling pathways that are regulated by MyD88 and TRIF [4]. Recently, it was established that CD14 is required for activation of the TLR4/TRIF pathway by either S-LPS or R-LPS [14]. TRIF-dependent signaling is essential for the expression of the majority of LPS-induced genes in macrophages [25], including IFN-α/β [26]. In line with others [27], we found in the present study that TRIF was required for LPS-induced secretion of TNF in the lung, but dispensable for the infiltration of the lung by PMN. Of interest, the release of LIX into BALF, which is considered to occur exclusively by respiratory epithelial cells [20], was not (R-LPS) or only modestly (S-LPS) influenced by the presence of TRIF. Considering that PMN influx in response to intrapulmonary administration of LPS largely depends on activation of the respiratory epithelium [24], these data together suggest that TRIF deficiency does not impact on the responsiveness of lung epithelial cells toward LPS in vivo.

A limitation of the present study is that the effect of LPS on cytokine release and neutrophil influx in the lung was studied at one time point only. Previously, we performed a kinetic analysis of LPS dosage effects in wild-type and LBP-deficient mice and found that both cytokine release and neutrophil influx in the lung peaked at 6 hours after LPS instillation, with the exception that neutrophil infiltration triggered by high dose LPS further increased at a later time point (22 h) [6]. Similar kinetics of cytokine release and neutrophil infiltration of the lung (with maximum responses at the 2 and 8 hour time point, respectively) after intranasal LPS instillation in wild-type and CD14-deficient mice were found by others [18]. On the basis of these results we have chosen to investigate the effect of LPS on both cytokine release and neutrophil influx in the lung only at the 6 hour time point. Further studies are required to determine the detailed kinetics of LPS dosage effects in wild-type and CD14-deficient mice.

In summary, our study shows that the effects of both S-LPS and R-LPS in the lung are mediated by pulmonary CD14. Acute lung inflammation induced by low doses S-LPS or R-LPS was dependent on CD14, whereas inflammatory responses induced by high LPS doses were diminished in the presence of CD14. Further studies are required to disentangle the dual role of CD14 in LPS-induced acute lung inflammation.

## Materials and Methods

### Ethics statement

The Animal Care and Use Committee of the University of Amsterdam approved all animal experiments. Experiments have been conducted according to national guidelines.

### Mice

Pathogen-free 10–12 week old WT mice (Harlan Sprague Dawley, Horst, Netherlands), TLR4KO, MyD88KO, CD14KO mice (Jackson Laboratories, Bar Harbor, ME) and TRIF mutant (TRIFmut) mice (all on C57BL/6 genetic background) were used in this study. Knock-out and mutant mice were generated as described previously [26], [28]–[30].

### LPS-induced lung inflammation

Lung inflammation was induced in mice as described previously [6]. *Salmonella abortus equi* S-LPS or *Salmonella minnesota* Re595 R-LPS (Alexis, San Diego, CA) was diluted at different doses (0.1 µg, 1 µg or 10 µg) in 50 µl sterile pyrogen-free 0.9% saline and instilled intranasally during anesthesia by inhalation of isoflurane (Abbott Laboratories, Kent, UK). Six hours after LPS inoculation, mice were anesthetized with ketamin (Eurovet, Bladel, Netherlands) and medetomidin (Pfizer, Capelle, Netherlands) and sacrificed by bleeding out the vena cava inferior. In separate experiments, CD14KO mice were treated intranasally with sCD14 (1 or 10 µg) and S-LPS (0.1 or 10 µg) simultaneously.

### Bronchoalveolar lavage

Bilateral bronchoalveolar lavage (BAL) with two 0.5-ml aliquots of sterile saline was performed as described previously [6]. Total cell numbers were counted using a Z2 Coulter counter (Beckman-Coulter, Miami, FL). BAL fluid (BALF) differential cell counts were performed on Giemsa-stained cytospin preparations. BALF supernatant was stored at −20°C until analysis.

### Mouse recombinant sCD14

Mouse recombinant sCD14 (AA16-336) lacking N- and C-terminal signal peptides was produced by cultured *Drosophila* S2 cells according to previously described procedures [31]. Briefly, sCD14 was amplified from lungs of WT C57BL6 mice using specific primers: 5′- AAAAAAAACCATGGTCTCCCGCCCCACCAGAG -3′ and 5′- AAAAAAAATCTAGAGTTAAACTTCTCCGAGTG -3′ and high fidelity Taq polymerase (Invitrogen, Carlsbad, CA), and subcloned into pMTBiP/V5-HisA (Invitrogen). Transfected S2 cells were induced with 500 µM copper sulfate for 4 days to secrete sCD14 in serum-free medium. Mouse recombinant sCD14 was purified from the culture supernatant using a HIS-trap nickel column (GE Healthcare Biosciences, Uppsala, Sweden). The purified 48-kDa protein enhanced LPS-induced TNF secretion by alveolar macrophages *in vitro* (data not shown). The endotoxin level was 0.8 EU/mg sCD14 as determined by limulus amoebocyte lysate assay (Lonza, Verviers, Belgium).

### Assays

BALF TNF, LPS-induced CXC chemokine (LIX, CXCL5) and sCD14 levels were measured using ELISA (TNF, LIX: R&D Systems, Minneapolis, MN; sCD14: Biometec, Greifswald, Germany). The detection limit was 15.6 pg/ml for TNF and LIX, and 3.13 ng/ml for sCD14.

### Statistical analysis

Data were analyzed using GraphPad Prism software version 4.03 (GraphPad Prism, San Diego, CA). The Mann-Whitney *U* test was used for calculating differences between two groups. All data are given as means ± SEM. P-values less than 0.05 were considered statistically significant.
